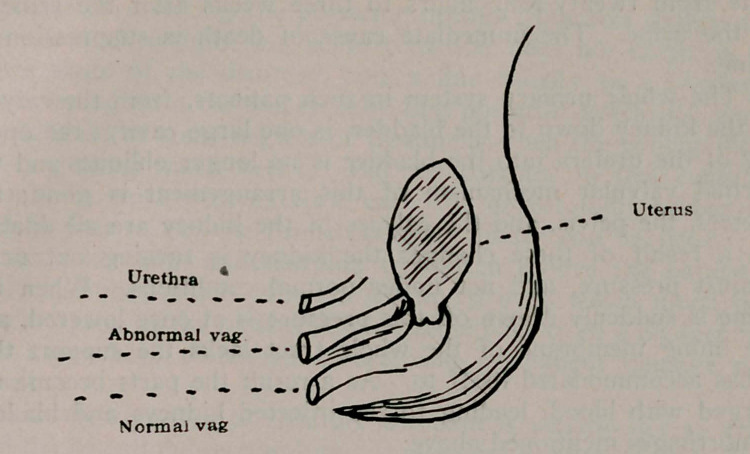# Double Vagina

**Published:** 1909-05

**Authors:** Irvin Hardy

**Affiliations:** A. B., M. S., F. S. Sc., Lond. Surgeon in charge, Allegheny Heights Hospital, Davis, W. Va.


### Double Vagina

By IRVIN HARDY, A. B., M. S., F. S. Sc., Lond.
Surgeon in charge, Allegheny Heights Hospital, Davis, W. Va.

ABOUT September 25, 1903, I was called to see Mrs. B., age 23, white, primipara. I found her in labor; pains severe, which were coming on at intervals of fifteen or twenty minutes. After the usual preparation of hands and rubber gloves, I proceeded to make a vaginal examination and found what I at first thought to be a misused urethra, but by passing index finger into canal I found it opened into the uterus. I could feel the presenting part of child, which was vertex. This canal or abnormal vagina opened into uterus just at upper limit of cervix. The normal vagina, which was below, was rather small. From below up their relation was, normal vagina, abnormal vagina, and urethra, as I shall show by diagram. The abnormal os was doing its best to dilate, so at first there was a question as to which vagina would afford passage for the child. Later on—about two hours—the normal os dilated and while pains were severe she was unable to complete the labor unassisted. I called my associate, Dr. R. Hardwick, and we proceeded to deliver with forceps, lacerating the perineum somewhat. The child was normal. Since that time she has had two normal births with no trouble.

My reason for reporting this case is that so far as I can learn there is no similar case of double vagina on record, *i.e.*, double vagina, single uterus; one vaginal canal directly over the other. If not, this should be made a matter of record.

## Figures and Tables

**Figure f1:**